# Unsafe Harbor? Elevated Blood Lead Levels in Refugee Children

**DOI:** 10.1289/ehp.121-a190

**Published:** 2013-06-01

**Authors:** Charles W. Schmidt

**Affiliations:** **Charles W. Schmidt**, MS, an award-winning science writer from Portland, ME, has written for *Discover Magazine*, *Science*, and *Nature Medicine*.

Two years ago, a doctor at Boston Children’s Hospital gave a routine physical to a 6-month-old baby boy who appeared to be wearing cosmetic eyeliner. His parents, recent immigrants from Nigeria, had been applying this folk remedy, known as *tiro*,^^1^^ under the baby’s eyes since he was 2 weeks old, believing it would improve his visual development.

The baby was later found to have a blood lead level (BLL) of 13 µg/dL, more than twice the reference level of 5 µg/dL at which the Centers for Disease Control and Prevention (CDC) recommends action to reduce exposure.^^2^^ Analysis later showed the *tiro* consisted of more than 80% lead. When the baby’s parents stopped using the product at the hospital’s urging, his BLL dropped to 8 µg/dL within three months.^^3^^

This anecdote illustrates a problem that persists despite bans on lead-based paint and leaded gasoline that have drastically reduced rates of elevated BLL among children. Overall, just 2.6% of U.S. children aged 1–5 years now have BLLs above the CDC reference level.^^4^^ In contrast, with BLLs often many times the national average, refugee children from developing countries constitute stubborn pockets of elevated BLLs in the United States.

Refugees are defined as individuals who have fled their home countries because of war, persecution, or the demonstrable threat of persecution. Their refugee status, for which they must apply, makes them eligible for medical and cash assistance within the United States.^^5^^ The CDC recommends that all refugees aged 6 months to 16 years be screened for BLL, anemia, and nutritional status upon arrival in the United States, with followup lead testing three to six months after placement in permanent housing.^^6^^

Lead screening is not mandated for immigrant children, who are not fleeing safety threats that could otherwise afford them refugee status. Because the CDC doesn’t report BLLs specifically by immigration status, the evidence for high BLLs in these groups comes mainly from a limited number of studies using refugee health screening data. However, much of what will be discussed in this story could be expected to apply to immigrant children as well.

The most recently reported lead poisoning fatality caused by lead-based paint in this country was a 2-year-old Sudanese girl, who died in 2000 in Manchester, New Hampshire, just weeks after her arrival from a refugee camp in Egypt. An autopsy showed a large increase in lead exposure in the preceding month. Among other potential routes of exposure, the child had been observed eating plaster from holes in a wall in her home. Testing showed the paint on the wall contained high levels of lead.^^7^^

**Figure:**
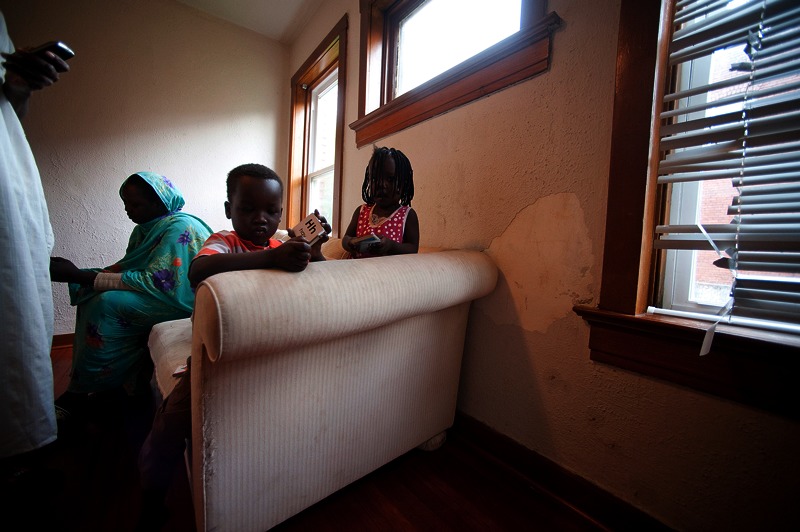
Sudanese refugees in their living room in Kansas City, Missouri. Although some refugees come to the United States with elevated blood lead, others are exposed only after their arrival. For these people, living in older housing with lead-based paint is often the cause of their exposure. Todd Feeback/Kansas City Star/MCT via Getty Images

“Refugee kids in particular can be malnourished and anemic, and that boosts lead absorption and heightens the potential for neurological effects,” says Mary Jean Brown, chief of the CDC’s Healthy Homes/Lead Poisoning Prevention Branch. She adds that refugee parents may have no idea about the risks posed by lead toxicity.

But with day-to-day survival a pressing concern, lead toxicity—especially if its effects aren’t outwardly apparent—can rank low on a refugee’s list of concerns, according to Rosemary Caron, an associate professor at the University of New Hampshire in Durham. “If there’s no rash or fever or something they can see, they tend not to worry about it,” she says.

## Assessing the Problem

Many refugee children come to the United States with elevated BLLs. According to Brown, many exposure sources contribute to those higher levels. Industrial metal smelting without adequate pollution controls and cottage industries that recycle lead–acid batteries are common in some developing countries. Lead-based paint is also a contributing factor, as is roadside contamination from leaded gasoline, which was only recently phased out in much of the developing world (Brown says it’s now the exclusive form of gasoline in just four countries: Afghanistan, Tunisia, Myanmar, and North Korea). Even where leaded gasoline is no longer used, high lead levels persist in the soil near highways, boosting risks of childhood exposure. What’s more, families from some developing countries routinely eat and prepare their meals at home on the floor, where children can be more easily exposed to lead-contaminated dust and tracked-in soil—a custom they may continue in the United States.

Once here, says Brown, exposures can persist for two main reasons: because of continued exposure via traditional customs and products like *tiro*, and because refugees, who are often poor, wind up living in older housing with flaking lead-based paint. “The CDC recommended [BLL screening for refugee children] because it is well known that refugee families will move to less expensive housing … shortly after resettlement,” says Paul Geltman, a pediatrician with Harvard Medical School and the Cambridge Health Alliance. “The idea is to capture those who may move into housing with lead hazards.”

Geltman says BLLs are higher on average in refugee children versus U.S.-born children, an assertion backed up by published reports from Massachusetts, New Hampshire, Minnesota, Indiana, Rhode Island, Florida, California, and other states. Geltman’s own published research shows, for instance, that 16% of refugee children who resettled in Massachusetts between 2000 and 2007 had BLLs in excess of 10 µg/dL (at that time the threshold for public health action),^^8^^ compared with 1.4% of U.S. children aged 1–5 years during the 1999–2004 time frame.^^9^^ And according to Geltman, unpublished findings based on data gathered from 1999 to 2009 by the Massachusetts Department of Public Health show that a whopping 23% of African refugee children (most of whom, at that time, were from Somalia) had BLLs exceeding 10 µg/dL. In the newest of these studies, published in January 2013, Brown and her CDC coauthors found that refugee children living in Manchester, New Hampshire (where more than 5,000 refugees have been resettled since 1990) were more than twice as likely as U.S.-born children to have BLLs above 10 µg/dL.^^10^^

Some refugees encounter lead hazards only once they are in the United States. CDC evidence shows that nearly 30% of 242 refugee children in New Hampshire developed elevated BLLs within three to six months of coming to the United States, even though their levels were not elevated during initial screening.^^11^^ Similarly, Geltman reported in 2011 that refugee children in Massachusetts were 12 times more likely to have a BLL over 20 µg/dL one year after an initial screening than nonrefugee children of the same age living in the same communities.^^8^^

**Figure:**
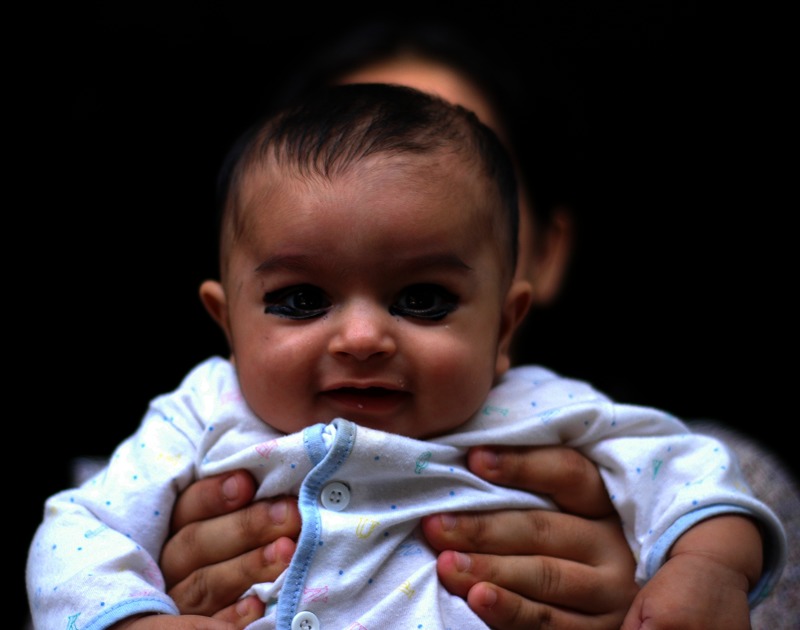
A Pakistani baby boy, his eyes lined with *kohl*. *Kohl* and similar products are believed to help children’s eyes develop as well as protect them against curses, but they can also expose wearers to lead. Educating refugees about the health risks involved requires diplomacy and sensitivity. © 2012 Ameer Hamza/Getty Images

Geltman says he was at first unsure as to what was causing the increases, but he had a hunch that lead-contaminated older housing was at least partly to blame. To investigate, he and coauthor Katherine Eisenberg, currently a resident in family medicine at the University of Rochester Medical Center, matched cases of rising BLL in refugees under age 7 with the median age of housing in Massachusetts. The results confirmed his hunch: Living in zip codes dominated by pre-1950s housing was associated with a 69% increase in the risk of a child’s BLL rising within 12–15 months of arrival.^^8^^

## Cultural Differences

At the same time, limited evidence suggests that imported traditional products add to that risk. In 2009, for instance, CDC investigators traced a cluster of 14 cases of elevated BLL among Burmese refugee children resettled in Indiana to a digestive folk medicine sold under the name Daw Tway. The medicine had a lead content of 520 ppm, and the children had BLLs ranging from 10.2 to 29.0 µg/dL, with a median value of 18.0 µg/dL.^^12^^

Tisha Titus, a physician at Federal Occupational Health in Atlanta, Georgia, says that Daw Tway often contains high levels of lead and, in some cases, arsenic. “We don’t know why, but we suspect the lead is added to the product,” says the CDC’s Brown, who explains the levels are typically too high to reflect background contamination.

Literally dozens of other lead-contaminated imported products have been documented in the United States, including lead glazing in Mexican pottery, lead-soldered cookware, and even contaminated candy from Latin America.^^13^^ According to Brown, lead ends up in these products for a variety of reasons. “In Ayurvedic medicines, we know that lead is intentionally added because it is thought to have medicinal properties,” she says. “Sometimes lead leaches into the candies from wrapping. Sometimes the ingredients are contaminated during processing. And lead is sometimes intentionally added to spices, teas, and other commodities sold by weight to boost prices.”

Last year, six pregnant Indian women who were living in New York City developed BLLs ranging from 16 to 64 µg/dL after taking a variety of Ayurvedic medications that were loaded with lead, arsenic, and mercury, according to a CDC report.^^14^^ In each case, the products were taken for issues related to pregnancy—one woman took them for nausea, several others to clear skin problems, and still another to improve her chances of having a boy. Prenatal lead exposures are particularly dangerous for the fetus; according to the CDC, they can limit fetal growth, cause neurodevelopmental problems, and heighten risks for premature birth and miscarriage.^^14^^

**Figure:**
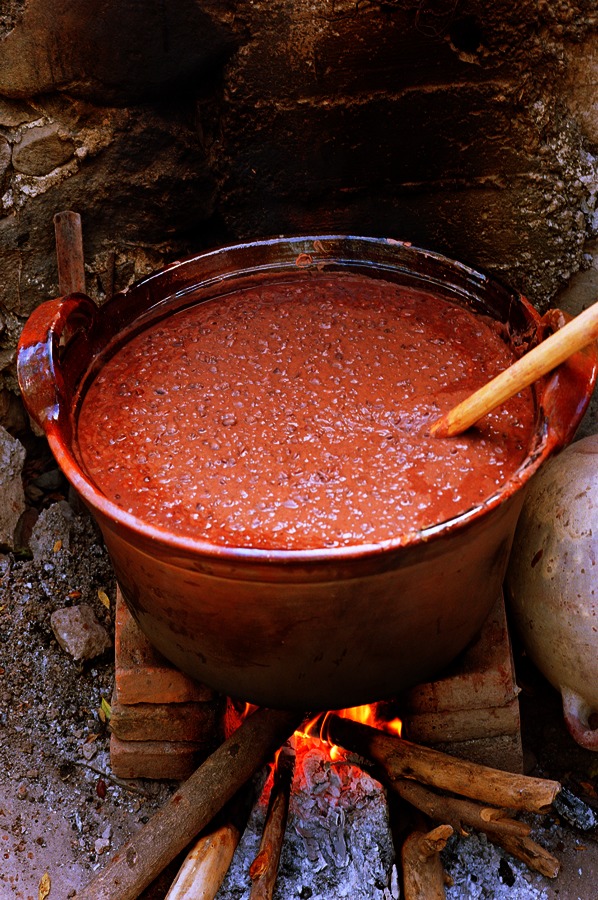
Traditional clay dishes like this Mexican pot often use lead glaze, which gives pottery a smooth finish and makes colors pop. It can also leach lead into foods. © Brooke Slezak/Getty Images

Still, the notion that these products are harmful can be challenging to convey between cultures. Referring to herbal remedies in particular, Titus says, “They’re based on recipes handed down for generations. So for a Western doctor to come in and say ‘what you’re doing can make your child sick’ isn’t going to sit well. You face a delicate balance of trying to maintain the integrity of the culture while at the same time providing a safer alternative.”

Based on research conducted in the Manchester community, Caron says that Western health workers working with African refugees confront language barriers, trust issues, and the fact that refugees can be overwhelmed by the resettlement process.^^15^^ As a white professional woman, Caron says she expects the African refugees she works with to view her with some initial skepticism. “If I pull up outside their homes in a minivan wearing a suit, they’re reluctant to even open the door,” she says.

To gain better access to these groups, Caron teamed up with Thandi Tshabangu-Soko, a colleague from Zimbabwe who immigrated to the United States 10 years ago. Now a Ph.D. candidate in health professions education at Simmons College in Boston, Tshabangu-Soko works with Caron on refugee health issues, particularly housing-based lead hazards from flaking paint.

Language barriers pose the biggest problem in communicating about lead risks, they have found. Plus, information about lead hazards may have been conveyed to refugees during the bewildering 24 hours after the family arrives at its new home, and they may not remember or comprehend the significance of what they’ve been told, Tshabangu-Soko says. For those refugees who don’t read or write English, she adds, written warnings won’t help.

## A Focus on Housing

In addition to connecting refugees with initial health assessment services, resettlement agencies holding contracts from the U.S. Department of State provide “wrap-around” reception and placement services that include housing arranged in advance. Under a provision of the Lead Disclosure Rule,^^16^^ landlords must reveal lead hazards and supply new tenants with a pamphlet called “Protect Your Family from Lead in Your Home,” which is published by the U.S. Environmental Protection Agency (EPA). The EPA publishes the pamphlet in several languages, but the U.S. Department of Health and Human Services’ Office of Refugee Resettlement (ORR) notes on its website that most landlords only have the English version.^^17^^ Another regulation known as the Lead-Safe Housing Rule^^18^^ requires that landlords who take federal funds for renting pre-1978 housing follow what ORR describes as “extremely complex” federal regulations aimed at addressing and containing lead hazards. The protections under the Lead-Safe Housing Rule vary depending on the type of public assistance used to cover housing costs.^^18^^

Individual states address lead threats in refugee housing with varying levels of stringency. Massachusetts has among the strictest requirements—refugee housing for children under age 6 years must be “lead-safe.”^^19^^ According to Brown, this means flaking lead-based paint must be controlled by repainting, while total removal is required only on “bitable” surfaces and those that are prone to rubbing or friction.

**Figure:**
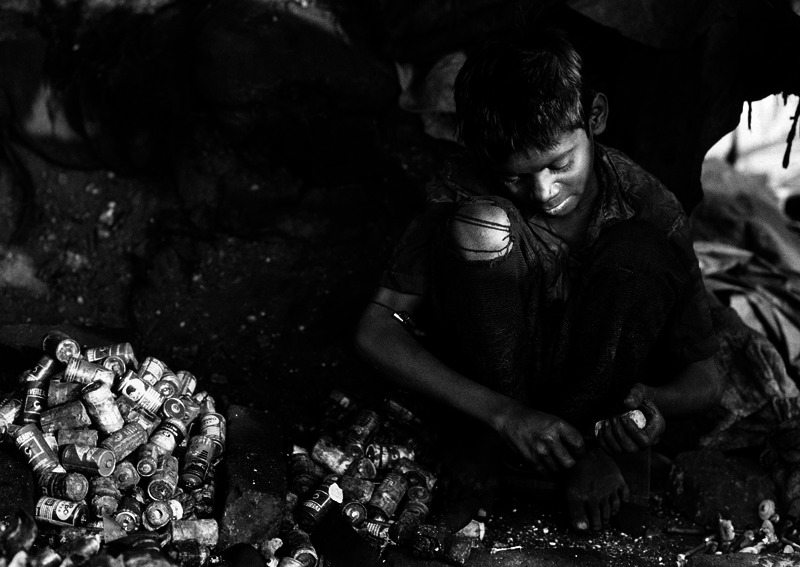
A Calcutta child working in a battery recycling shop. Shops like this are common in some developing countries—and a common source of lead exposure prior to emigration. © Philippe Lissac/Godong/Corbis

But according to Geltman, the regulations aren’t always followed. In some cases, refugees wind up in lead-contaminated housing either because resettlement agencies aren’t aware of the risk in a given dwelling or because landlords don’t reveal it. Several years ago, Massachusetts officials visited resettlement agencies throughout the state with a reminder that refugee dwellings should be lead-safe, and preferably lead-free if they house children or women of child-bearing age.

Jennifer Cochran, director of the Division of Global Populations and Infectious Disease Prevention at the Massachusetts Department of Public Health, says the State Department’s financial support for refugees lasts three months at most. Faced with dwindling support, refugees will likely try to lower expenses by looking for cheaper accommodations with even greater—and comparatively unmonitored—lead risks. Because refugee movements in Massachusetts aren’t actively tracked post-resettlement, the added lead risk that comes from these subsequent housing choices isn’t well characterized. However, Cochran points back to the evidence from Geltman and Eisenberg’s 2011 report (which she coauthored) indicating refugee children were more likely to develop elevated BLLs than same-age children in the same high-risk communities.^^8^^

## Next Steps

Government groups are now confronting the problem of refugee lead hazards on both domestic and international fronts. The CDC’s lead screening recommendations for refugees are part of that strategy, Brown says. She cites evidence^^10^^ showing that implementation of the recommendations was associated with a reduction in the time it took for individuals’ BLLs to drop below 10 µg/dL. “Early identification and appropriate followup make BLLs come down faster,” she explains. U.S. agencies, including the CDC and the EPA, are also working with the World Health Organization and the United Nations Environment Programme on its Global Alliance to Eliminate Lead in Paints program, she says. That program’s broad objective is to phase out the manufacture and sale of lead-based paint worldwide.

**Figure:**
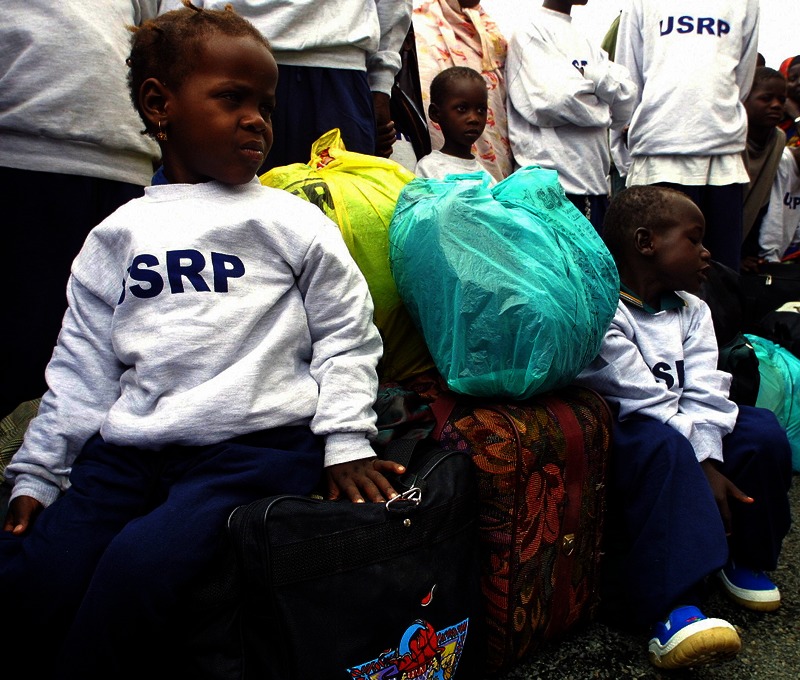
Somali Bantu children in shirts provided by the U.S. Refugee Program, waiting for a bus en route to new homes in the United States. Many refugees arrive in the United States overwhelmed and concerned about day-to-day survival. Lead toxicity—especially if its effects aren’t outwardly apparent—can rank low on their list of concerns. Simon Maina/AFP/Getty Images

According to Cochran, Massachusetts resettlement workers coordinate with bilingual community health workers who emphasize key talking points related to healthy homes. “We talk about bringing food preparation up off the floor and why it’s important to use a table for food, and to watch where kids play,” she says. “But lead risks can be difficult to grasp for refugees, who are often coming from very dangerous situations to the safety of the United States. The notion that here your house or a traditional medication could poison you makes little sense.” Many refugees arrive in the United States believing that everything in this country is great, and they will be safe, she adds, “so this becomes part of the larger conversation that goes with taking care of yourself here.”
