# Erratum: “Unsafe Harbor? Elevated Blood Lead Levels in Refugee Children”

**DOI:** 10.1289/ehp.121-a240

**Published:** 2013-08-01

A passage in the June 2013 News article “Unsafe Harbor? Elevated Blood Lead Levels in Refugee Children” [Environ Health Perspect 121:A190–A195 (2013)] attributed provisions of the Lead-Safe Housing Rule to the Lead Disclosure Rule, and certain details of both rules were omitted or unclear. The article also credited the statement that Massachusetts has some of the strictest requirements for lead in housing to the U.S. Department of Housing and Urban Development instead of to the Massachusetts Department of Public Health. 

It should be noted that, although this story focused on renters and landlords, the Lead Disclosure Rule also applies to buyers and sellers of pre-1978 housing.

*EHP* regrets the errors.

